# Identification of a Major QTL (*qRRs-10.1*) That Confers Resistance to *Ralstonia solanacearum* in Pepper (*Capsicum annuum*) Using SLAF-BSA and QTL Mapping

**DOI:** 10.3390/ijms20235887

**Published:** 2019-11-23

**Authors:** Heshan Du, Changlong Wen, Xiaofen Zhang, Xiulan Xu, Jingjing Yang, Bin Chen, Sansheng Geng

**Affiliations:** Beijing Vegetable Research Center (BVRC), Beijing Academy of Agriculture and Forestry Sciences, National Engineering Research Center for Vegetables, Beijing Key Laboratory of Vegetable Germplasm Improvement, Beijing 100097, China; duheshan@nercv.org (H.D.); wenchanglong@nercv.org (C.W.); zhangxiaofen@nercv.org (X.Z.); xuxiulan@nercv.org (X.X.); yangjingjing@nercv.org (J.Y.)

**Keywords:** *Capsicum annuum*, *Ralstonia solanacearum*, SLAF-seq, QTL, linkage mapping, rootstock

## Abstract

The soilborne pathogen *Ralstonia solanacearum* is the causal agent of bacterial wilt (BW), a major disease of pepper (*Capsicum annuum*). The genetic basis of resistance to this disease in pepper is not well known. This study aimed to identify BW resistance markers in pepper. Analysis of the dynamics of bioluminescent *R. solanacearum* colonization in reciprocal grafts of a resistant (BVRC 1) line and a susceptible (BVRC 25) line revealed that the resistant rootstock effectively suppressed the spreading of bacteria into the scion. The two clear-cut phenotypic distributions of the disease severity index in 440 F2 plants derived from BVRC 25 × BVRC 1 indicated that a major genetic factor as well as a few minor factors that control BW resistance. By specific-locus amplified fragment sequencing combined with bulked segregant analysis, two adjacent resistance-associated regions on chromosome 10 were identified. Quantitative trait (QTL) mapping revealed that these two regions belong to a single QTL, *qRRs-10.1*. The marker ID10-194305124, which reached a maximum log-likelihood value at 9.79 and accounted for 19.01% of the phenotypic variation, was located the closest to the QTL peak. A cluster of five predicted *R* genes and three defense-related genes, which are located in close proximity to the significant markers ID10-194305124 or ID10-196208712, are important candidate genes that may confer BW resistance in pepper.

## 1. Introduction

The soilborne pathogen *Ralstonia solanacearum* is the causal agent of bacterial wilt (BW), a disease that leads to severe global crop losses in the family *Solanaceae* [1,2,3,4]. The pathogen infects the host by penetrating the root epidermis via natural openings or wounds, and crosses the cortex and endodermis to finally reach the root xylem. In the xylem, it starts extensive colonization and spreads to the aerial parts of the infected plant along the vascular system, thus blocking the water flux from root to shoot, finally, causing severe wilt symptoms [3,5]. Diverse chemical and biological control strategies have been applied to control this devastating disease, but have proved ineffective [6,7]. 

BW is widely prevalent in peppers across much of Asia [1,4,8,9,10]. Resistance breeding is considered the best strategy for the management of this devastating soilborne disease [11]; however, only a few pepper lines are resistant to BW. Resistant accessions identified in earlier studies were mainly small-fruited types, such as LS2341, Manganji, White Khandari, Plant C-1, and PM687 [12,13,14,15]. Recently, we found that the elite inbred pungent pepper line BVRC 1 is highly resistant to *R. solanacearum* strain Rs-SY1. Bioluminescence and colony-forming unit scoring assays indicated that pathogen multiplication is restricted within the roots, and spreading of the pathogen in the aerial stem is limited in this line [1]. The identification of new resistant lines would broaden the genetic basis for the development of pepper cultivars with improved disease resistance in future. Curiously, root and collar colonization by *R. solanacearum* after root-dip inoculation is not prevented in BVRC 1, leading to questions about the host–pathogen colonization dynamics in graft combinations of resistant and susceptible peppers. 

Genetic inheritance of BW resistance in pepper has been studied in progeny studies and by quantitative trait locus (QTL) analysis. Resistance is generally quantitatively inherited and is controlled by at least two genes in the sweet pepper cultivar Mie-Midori [16] and by two to five genes with additive effects in the pepper line PM687 [13]. Resistance to *R. solanacearum* strain KP9547 in the pepper line LS2342 reportedly is polygenic, and is linked to a major QTL named *Bw1* on chromosome 1 [17]. Diverse modes of gene interaction that depend on the virulence level of *R. solanacearum* isolates have been reported in peppers, indicating the complexity of the inheritance of BW resistance [10]. Molecular genetic analysis of an F_2_ population constructed from near-isogenic lines (Anugraha and Pusa Jwala) revealed that BW resistance is recessive, and polymorphic AFLP marker (*Eco*ACT + *Mse*CAC) bands (103, 117, and 161 bp) associated with the recessive resistant allele have been identified [18]. The tomato cultivar Hawaii 7996 has stable resistance to *R. solanacearum*, and is intensively used for BW resistance QTL mapping. Two major QTLs, named *Bwr-6* and *Bwr-12,* located on chromosomes 6 and 12, respectively, are reported to be closely associated with resistance to BW [19,20,21,22]. Recently, using genome-wide SNP analysis, Kim et al. [23] indicated that *Solyc12g009690.1*, encoding a putative leucine-rich repeat (LRR) receptor-like protein, might be tightly linked to the major BW resistance QTL *Bwr-12* in tomato. Additionally, QTLs on chromosomes 3, 8, 10, and 11 were also reported and may be important for field BW resistance in tomato [20]. 

QTL mapping is a critical approach for the genetic dissection of quantitative traits, and tightly linked markers can be used in marker-assisted selection (MAS) in plant breeding. Traditionally, QTL mapping involves the genotyping of a large number of individuals in segregating populations; however, this is labor-intensive, time-consuming, and costly. Bulked segregant analysis (BSA) is a simplified strategy to rapidly identify markers linked to target genes or QTLs, affecting a trait of interest based on genotyping of only a pair of pooled DNA samples from two sets of individuals with distinct or opposite extreme phenotypes [24]. Compared with traditional QTL mapping, BSA does not require the laborious process of identifying the trait value of each individual, but requires only the identification of individuals that exhibit opposite extreme phenotypes, and is less sensitive to occasional phenotyping mistakes [25]. However, it is challenging to develop thousands of candidate molecular markers and screen them in bulked pools to discover a small subset of markers for diagnosing the target phenotype. Next-generation sequencing (NGS) has allowed for the development of new strategies to leverage the advantages of BSA. Numerous methodologies and applications of high-throughput genotyping-assisted BSA—including MutMAP, QTL-seq, and SHOREmap—for the detection of major QTLs for complex plant traits or the physical locations of mutations have been reported [25,26,27]. Specific-locus amplified fragment sequencing (SLAF-seq) is a strategy used to discover single-nucleotide polymorphisms (SNPs), facilitated by reduced-representation genome sequencing and NGS technologies [28]. The combination of SLAF-seq and BSA technologies (SLAF-BSA) has been successfully used for the identification of major QTLs linked to specific traits in plant species [29,30,31]. For example, in pepper, SLAF-BSA has been successfully used for the identification of major QTLs for first flower node [32], and resistance to *Phytophthora* root rot [33] and cucumber mosaic virus [34].

Information on markers linked with BW resistance in pepper remains scant, limiting the use of MAS in resistance breeding against this disease. Therefore, this study aimed to (1) monitor the bacterial infection dynamics in reciprocal grafts of the resistant line BVRC 1 and the susceptible line BVRC 25 by using the bioluminescent *R. solanacearum* strain BL-Rs7; (2) use integrated SLAF-BSA strategies to rapidly identify regions associated with BW resistance; and (3) perform QTL mapping to verify the regions associated with and identify markers linked to BW resistance in pepper. 

## 2. Results

### 2.1. Visualization of Bioluminescent R. solanacearum Colonization of Grafted Plants

**Figure 1:**
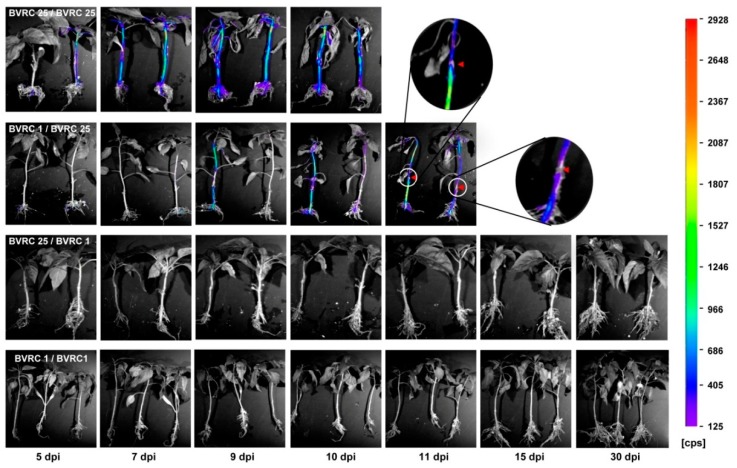
Real-time visualization of the bacterial infection dynamics in reciprocal grafts of a resistant (BVRC 1) and a susceptible (BVRC 25) line after root-dip inoculation with the bioluminescent *R. solanacearum* strain BL-Rs7. Scion/rootstock combinations are indicated. The red triangles indicate the position of the graft union. Bioluminescence imaging of the infected plants was conducted using a Berthold NightSHADE CCD camera. The color-coded bar displays the bioluminescence intensity scale.

Grafting experiments revealed that the resistant line BVRC 1 had a good scion/rootstock compatibility with the susceptible line BVRC 25. The average graft success rates of BVRC25/BVRC1 and BVRC1/BVRC25 were 90.2% and 85.9%, respectively. Typical disease development in the four graft combinations after root-dip inoculation with the bioluminescent *R. solanacearum* strain BL-Rs7 is shown in Figure 1 and Figure S1. Strong luminescence signals were detected in the stems of the BVRC 25/BVRC 25 plants at 5 days post inoculation (dpi) and increased gradually overtime, leading to whole plant death within 10 dpi. Neither wilting nor luminescence signals were detected in 94.6% of the stems of the BVRC 1/BVRC 1 plants from 5 to 30 dpi (Figure 1 and Figure S1). *R. solanacearum* multiplication in the scions of BVRC 1/BVRC 25 grafted plants was conspicuously retarded until 9 dpi when compared with that in BVRC 25/BVRC 25 plants, indicating that *R. solanacearum* multiplication in the stem of BVRC 1 was limited, but not completely suppressed. Interestingly, the bioluminescence intensity at the graft union of the BVRC 1/BVRC 25 seedlings was consistently lower than that in the nearby rootstock and scion stems. More than 90.9% of the BVRC 25/BVRC 1 seedlings did not show signs of wilting and showed no luminescence signal in the stems, and their mortality rate did not significantly differ from that of BVRC 1 self-grafted plants (Figure S1). These results corroborated that the resistant line BVRC 1 can be used as a rootstock to manage bacterial wilt in pepper.

### 2.2. BW Resistance Inheritance in the Pepper Line BVRC 1

**Figure 2:**
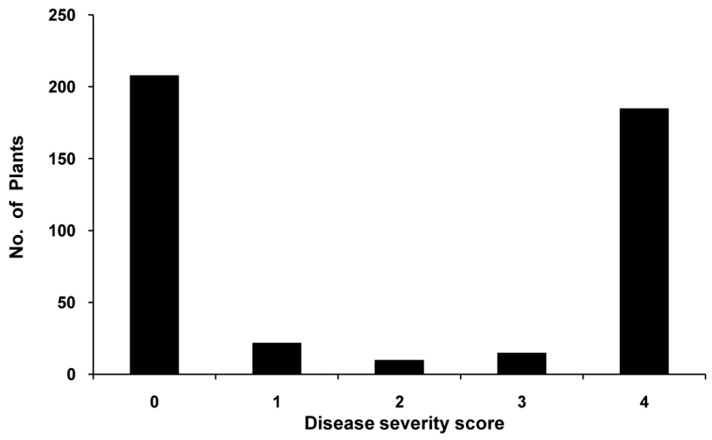
Histograms of the disease severity scores (ranging from 0 = asymptomatic to 4 = dead) of the F2 population (*n* = 440) in response to root-dip inoculation with *R. solanacearum* strain Rs-SY1 at 20 days post-inoculation.

The two parental lines, BVRC 1 and BVRC 25, clearly displayed differential reactions to root-dip inoculation with *R. solanacearum* strain Rs-SY1. The resistant parent BVRC 1 had a low wilt rate (4.58%), disease severity index (DSI) (0.31), and area under the disease progress curve (AUDPC) (1.68) when compared to the susceptible parent BVRC25 (100%, 3.98, and 42.92, respectively) (Table 1). The mid-parent wilt rate (52.29%) was close to that of the non-segregating population (F_1_) (48.59%), greater than that of the segregating population BC_1_P_r_ (24.94%), and lower than that of the segregating population BC_1_P_s_ (71.97%) (Table 1). Furthermore, wilting progressed conspicuously slower in dead F_1_ plants than in the susceptible parent, and the mean AUDPC value for dead F_1_ plants (29.32 ± 4.03) was lower than that for the susceptible parent (42.92 ± 1.84). These results suggested that BW resistance in BVRC 1 might be partially or incompletely dominant. In the 440 plants of the F_2_ population, the number of plants with susceptibility symptoms increased with increasing dpi. There were two clear-cut disease-response phenotypes in the F_2_ population at 20 dpi: asymptomatic or death (Figure 2), indicating that a major genetic factor as well as a few minor factors control BW resistance in BVRC 1. 

### 2.3. Association Analysis Based on Euclidean Distance (ED) and SNP Index

**Figure 3:**
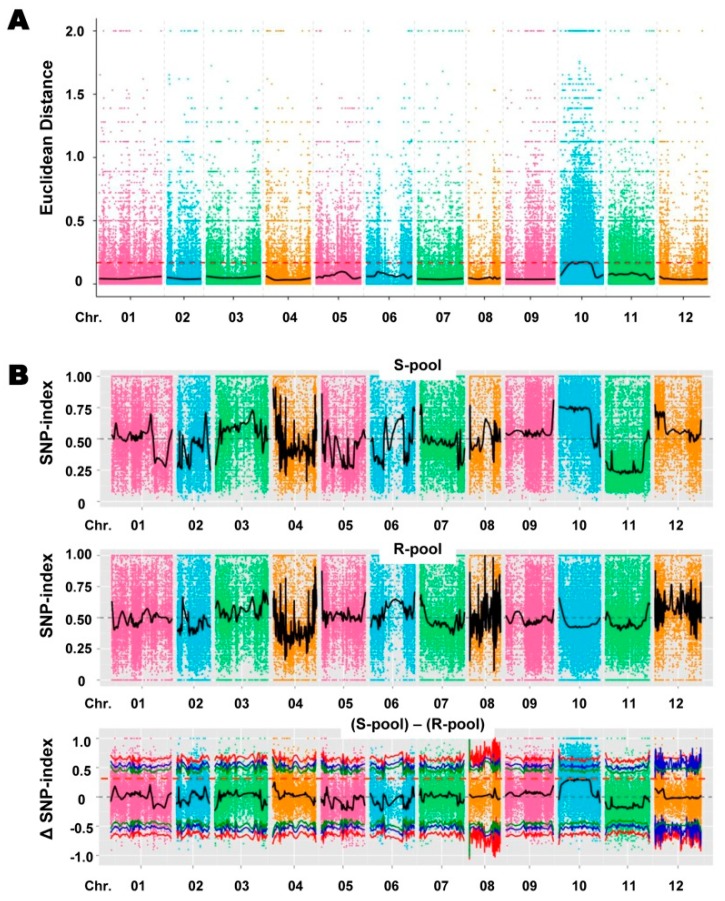
Identification of genetic region(s) significantly associated with resistance to *R. solanacearum* strain Rs-SY1 in pepper (*C. annuum*) using combined specific-locus amplified fragment sequencing (SLAF-seq) and bulked segregant analysis (BSA). (**A**) Euclidean distance (ED)-based association results. The *x*-axis represents the 12 pepper chromosomes, and the *y*-axis represents the association values based on the ED at each SNP location. The red dashed line represents the association threshold. Higher association values based on the ED indicate a stronger association between the SNPs and bacterial wilt resistance. (**B**) SNP index graphs of the R- and S-pools, and the Δ(SNP index). The x- and y-axes indicate the 12 pepper chromosomes and the SNP index, respectively. The red dashed line represents the association threshold.

SLAF-seq of the two parents and the resistant (R and susceptible (S pools yielded approximately 82.34 million raw reads. After filtering, 79.14 million clean reads were retained (Table S2). The average percentage of reads with a Q30 score was 92.89%, indicating that most reads were of high quality. An average of 97.59% of the clean reads from each of the four SLAF libraries could be mapped to the CM334 reference genome (Table S2). Cluster analysis identified 376 860 SLAFs that were distributed evenly over the chromosomes of the reference genome (Table S3; Figure S2). The average SLAF-seq depth was 23.20-fold in the BVRC 1 library, 26.46-fold in the BVRC 25 library, 63.76-fold in the R-pool library, and 67.06-fold in the S-pool library (Table S2). In total, 1,073,404 SNPs were identified using the GATK and SAMtools software [35,36]. The number of SNPs on each chromosome ranged from 42,077 on chromosome 8 to 147,987 on chromosome 9 (Table S3). After filtering, 161,389 polymorphic SNPs between the two DNA bulks were retained as useful markers for association analysis to identify candidate regions associated with BW resistance in pepper, using ED [37] and SNP index [26] methods. For the ED method, the marker–trait association threshold was set to 0.17, and several small candidate regions related to BW resistance were detected on chromosome 10 (Figure 3A). As the resistance response to BW might be partially dominant inheritance, the upper limit Δ(SNP index) value of the trait-associated SLAF is expected to be less than 0.5. Using Loess regression fitting (see Materials and Methods), the Δ(SNP index) threshold value for correlation between the R- and S-pools was set at 0.33, and numerous small candidate regions were identified in the similar regions of chromosome 10 as those identified by the ED method (Figure 3B). Finally, two large candidate regions on chromosome 10, including the intervals 56,910,000–69,110,000 and 111,090,000–183,670,000 (Table 2), which covered all the candidate regions from the two association methods, were considered to be potentially associated with BW resistance.

### 2.4. Construction of a Genetic Linkage Map of Chromosome 10

To confirm the BW resistance-associated regions identified by SLAF-BSA, 46 markers showing polymorphism between the parent lines and evenly distributed on chromosome 10, including 30 InDel and 16 SNP markers (Table S1), were genotyped in the 440 plants of the F_2_ population. The linkage map had a total length of 133.3 cm, with an average intermarker distance of 2.6 cm (Table S1). SNP10-64571038 was positioned the closest to the right border of the first association region (56 910 000–69 110 000), and ID10-117045647 was located the closest to the left border of the second association region (111,090,000–183,670,000). The physical distance between SNP-64571038 and ID10-117045647 was ~52.47 Mb based on the reference genome CM334, but the genetic distance in the linkage map was only ~1.3 cm. The ordering of some markers on the linkage map were not the same as their physical orders on reference genome. For example, maker ID10-61856194 developed in the first associated region was mapped between the makers ID10-134713789 and ID10-142883512, which were both physically distributed in the second associated region (Figure S3 and Table S1). We inferred that a part of associated SLAFs from first associated region may be assigned to the second associated region. Therefore, these two adjacent regions may form one QTL. 

### 2.5. Confirmation of the BW Resistance-Associated Regions by QTL Mapping

**Figure 4:**
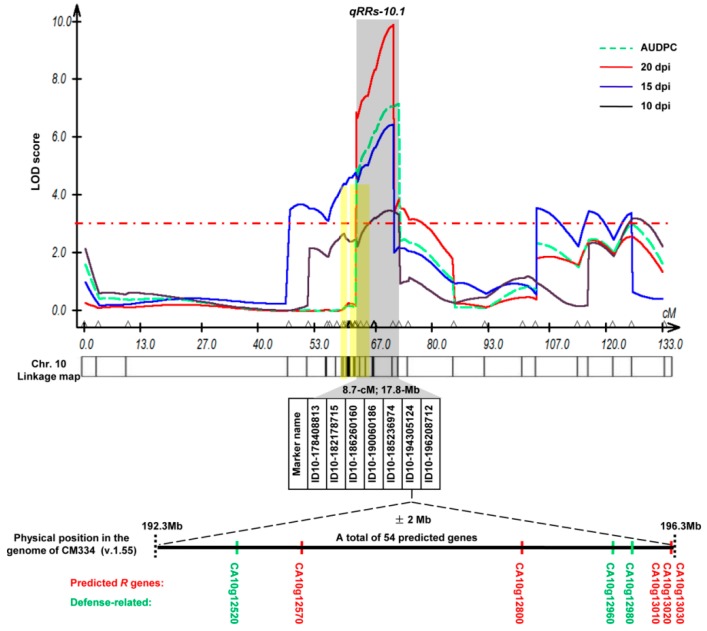
Quantitative trait locus (QTL) mapping for the identification and validation of *R. solanacearum* resistance QTL *qRRs-10.1* on pepper chromosome 10. An average 1000 permutation threshold (maximum log-likelihood = 3.0) is indicated by the dotted line. The above QTL graph was the original graph generated by WinQTL cartographer with the following modification. A QTL involved in each resistance component (DSI at 15 and 20 dpi, and AUDPC) was mapped to an overlap region, indicated with the gray bar. The approximate positions of the two resistance-associated regions identified by SLAF-BSA are indicated by the yellow bars. The bar below the QTL graph represents the genetic linkage map constructed with 46 InDel and SNP markers on chromosome 10. InDel markers within the resistance QTL *qRRs-10.1* are shown. The five predicted resistance (*R*) genes and three defense-related genes are shown in red and green, respectively.

Composite interval mapping (CIM) was used to identify BW resistance QTL(s) based on four resistance components, including DSI at 10, 15, and 20 dpi, and the AUDPC values (Figure 4). A significant QTL with a log-likelihood (LOD) score of more than 3.0 was detected on chromosome 10 as early as 10 dpi. QTL mapping based on the DSI value at 15 dpi revealed a large regional and significant QTL region that covered the two associated regions identified by SLAF-BSA. Based on the DSI at 20 dpi, the QTL reached a maximum plateau LOD score of 9.79 and accounted for 19.01% of the phenotypic variation. The significant QTLs involved in each resistance component were mapped to similar genome regions. Although the position of the QTL peak varied, the intervals overlapped for DSI at 15 and 20 dpi, and AUDPC, and this QTL was considered the major QTL for BW resistance in the pepper line BVRC 1 (Figure 4). The overlapping QTL region (designated “*qRRs-10.1*”) was mapped at 63.6–72.3 cm—an interval of 8.7 cm—between two InDel markers (ID10-178408813 and ID10-196208712) that were physically located in the region of 178.4–196.2 Mb (~17.8 Mb) on chromosome 10. Genetic mapping information of the QTL regional markers is provided in Table 3. Seven InDel markers were located within the major QTL *qRRs-10.1*, and ID10-194305124 was the closest to the QTL peak. Moreover, based on significant marker effects in the F_2_ population, ID10-194305124 showed the strongest correlation with *R. solanacearum* resistance when compared with the other markers, as F_2_ plants harboring the resistance allele of ID-194305124 showed the lowest mean AUDPC (6.36), and those harboring the susceptibility allele showed the highest AUDPC (35.52) (Table 3). The additive effect of the associated markers indicated that the BW resistance-increasing allele was contributed by the resistant parent BVRC1. The DR was 0.2–0.8 for all seven significant markers, indicating partial dominance of *qRRs-10.1*. Using the package R/qtl, it was verified that only one significant QTL peak existed on chromosome 10, and markers ID10-194305124 and ID10-196208712 were located the closest to the QTL peak (Figure S4). Thus, SLAF-BSA combined with QTL mapping identified a major BW resistance QTL, *qRRs-10.1*, on chromosome 10 of the pepper line BVRC 1.

### 2.6. Annotated Resistance-Related Genes and SNPs within the qRRs-10.1 Locus

In total, 54 genes were predicted to be closer than ±2 Mb from the highest significant maker ID10-194305124 (Table S4). Among these, five genes (CA10g12570, CA10g12800, CA10g13010, CA10g13020, and CA10g13030) were predicted to be putative disease resistance (*R*) genes. To characterize the metabolic pathways these 54 predicted genes are involved in, functional classification was conducted based on Gene Ontology (GO) term and Kyoto Encyclopedia of Genes and Genomes (KEGG) pathway analyses. Based on GO term enrichment analysis, two genes (CA10g12960 and CA10g12980) were assigned to “defense response” (GO: 0006952). KEGG analysis identified one gene (CA10g12520) involved in “plant–pathogen interaction” (ko: 04626). CA10g12960 and CA10g12980 are upregulated late in response to *Hyaloperonospora parasitica* (*LURP*) genes. CA10g12520 is a pathogenesis-related protein (*PR-1*) (Table 4 and Table S4). These three defense-related genes have been verified to be involved in plant–pathogen interactions [38,39]. Therefore, these eight resistance and defense-related genes are possibly important candidate genes for *qRRs-10.1*.

Alignment of the resequencing reads of BVRC 1 and BVRC 25 against CM334 identified 4840 SNPs among the regions of the 54 predicted genes, 221 of which were non-synonymous, stop-gained, or stop-lost SNPs located in the coding regions of 44 of the predicted genes (Table S5). One to eight non-synonymous SNPs were identified in the coding regions of each of the three *R* genes (CA10g13010, CA10g13020, and CA10g13030) and the two defense-related genes (CA10g12960 and CA10g12980) (Table 4 and Table S5). 

## 3. Discussion

### 3.1. BVRC 1 Is a Good Rootstock for BW Resistance

Grafting is widely applied in pepper production to improve plant vigor and yield and to provide resistance to soilborne pathogens and nematodes [40,41]. Our previous study on the dynamics of *R. solanacearum* colonization in planta revealed that bacterial populations in the root and collar of the resistant line BVRC 1 were smaller than those in the susceptible line BVRC 25 [1]. To further dissect rootstock control of BW resistance in BVRC 1, disease establishment was monitored and, simultaneously, the in planta bacterial infection dynamics were evaluated in reciprocal grafts of BVRC 1 and BVRC 25, as well as in self-grafted plants. By tracking bacterial invasion in the graft tissues of BVRC 1/BVRC 25, it was verified that *R. solanacearum* can cross the graft union (~2 cm up the cotyledons) from the rootstock to the scions; however, wilt onset was delayed compared to that in BVRC 25/BVRC 25 plants. More than 90.9% of BVRC 25/BVRC 1 plants did not show bioluminescence signals or wilting until flowering, indicating that the grafting onto the resistant BVRC 1 rootstock effectively suppressed the spreading of *R. solanacearum* into the scion. Our previous [1] and current studies revealed that BW resistance in BVRC 1 is associated with the restriction of *R. solanacearum* multiplication in the roots and lower stem. In vascular wilt diseases, resistance is generally considered to be a state in which pathogen growth in the aerial parts is substantially limited, although the pathogen is not prevented from infecting the roots [1,42,43,44]. In line with our results, analysis of the systemic spreading of *R. solanacearum* in *Medicago truncatula* revealed that in a resistant line, the bacterial load was reduced in the aerial tissues, but not the root tissues, suggesting that the hypocotyls and lower stem may be critical zones for suppressing bacterial growth [42,44]. Therefore, grafting of the resistance rootstock of BVRC 1 above the cotyledon and keeping the graft union away from the soil line might contribute to BW resistance in grafted plants. 

### 3.2. Like Most BW-Resistant Pepper Lines, BVRC 1 Has Partially Dominant Resistance

Genetic analysis of BW resistance and the identification of resistance genes are important in pepper breeding. Several sources of genetic resistance to BW have been identified in pepper to date. However, the genetic basis of BW resistance in these pepper lines remains elusive and is even controversial as different sources have different inheritance mechanisms [10,12,13,14,15,16,18]. In this study, comparison of the DSI values of F_1_, BC_1_P_s_, and BC_1_P_r_ with those of the parents indicated that BW resistance is a partially dominant trait (Table 1). Similarly, in previous studies, the disease severity in F_1_ was similar to or less than the mean mid-parent value [10,16], indicating that BW resistance in pepper generally is partially dominant. Conversely, Thakur et al. [18] studied the inheritance of BW resistance in an F_2_ population derived from two pepper near-isogenic lines, Pusa Jwala and Anugraha, which differ only in BW resistance, and found that the resistance was governed by homozygous recessive gene action. These discrepancies may be due to various factors, such as the use of different breeding lines, isolates, and the criteria used for scoring.

### 3.3. qRRs-10.1 Is a Partially Dominant Major Resistance QTL on Chromosome 10 of the Pepper Line BVRC 1

The phenotypic distributions for DSI and AUDPC were continuous, with a bimodal trend in the F_2_ population, indicating that a major genetic factor as well as a few minor factors control BW resistance in BVRC 1 (Figure 2). BSA can used to analyze quantitative traits that are controlled by a few major genes [45]. Thus, SLAF-BSA was utilized to rapidly identify candidate regions harboring QTLs for BW resistance. Only two adjacent associated regions (Table 2) were identified on chromosome 10, using both ED [37] and the SNP index [26]. The credibility of the first associated region was questionable because the marker ID10-61856194 derived from the first associated region was mapped in the second associated region based on the genetic linkage map. Additionally, QTL mapping revealed that only one significant QTL peak was identified near the second associated region (Figure 4). Therefore, these two regions may belong to a single QTL. QTL mapping showed that a common QTL, *qRRs-10.1*, identified based on four resistance components, was delimited between the markers ID10-178408813 and ID10-196208712, within ~17.8 Mb. The DR values of the significant markers within the QTL and the DSI values for F_1_, BC_1_P_s_, and BC_1_P_r_ compared with those of the parental lines indicated the partial dominance of the resistance phenotype for *qRRs-10.1* (Table 1 and Table 3). ID10-194305124 had the highest LOD score and phenotypic variation *R^2^* among markers at the *qRRs-10.1* locus (Table 3), demonstrating its potential use for MAS of BW resistance with BVRC 1 as the resistant donor parent. The major BW resistance QTL *qRRs-10.1* identified in the present study differs from those identified in previous studies that suggested the existence of disease resistance loci on chromosomes 1 and 11 [17,18]. This can be explained by the use of different parents, isolates, inoculation methods, scoring methods, and population types. Although a major QTL on chromosome 10 was rapidly identified and verified using SLAF-BSA combined with QTL mapping, we did not find any additional resistance-associated regions in other chromosomes. This may be because BSA is not useful for mapping minor QTLs [46]. Currently, 162 F_7_ recombinant inbred lines from a cross between BVRC 25 and BVRC 1 are being used to fine map *qRRs-10.1* and identify additional loci linked to BW resistance. 

### 3.4. qRRs-10.1 Candidate Genes

A growing number of *R* genes have been cloned and characterized. Most of them encode a predicted protein with an LRR domain and other conserved domains, such as a nucleotide-binding (NB) site [47,48,49]. Two *R. solanacearum* resistance genes have been identified in *Arabidopsis thaliana* through map-based cloning: *ERECTA*, encoding an LRR receptor-like kinase involved in polygenic resistance [50], and *RRS1-R*, encoding a typical TIR-NB-LRR protein with a WRKY domain involved in monogenic resistance and tolerance [51,52]. The *RRS1-R* gene and other adjacent NB-LRR gene *RPS4* function as a dual resistance gene system to prevent infection by three distinct pathogens, namely *R. solanacearum*, *Pseudomonas syringae*, and *Colletotrichum higginsianum* [53,54]. The RPS4/*RRS1-R* complex recognizes type III effector (T3E) *PopP2* from *R. solanacearum* via interaction with the RRS1-R WRKY domain, resulting in the activation of subsequent immunity [55,56]. In *M*. *truncatula*, the *R* locus *MtQRRS1* contains a cluster of seven *R* genes and may act through intralocus interactions to promote resistance to *R. solanacearum* [42]. Therefore, we aimed to identify *R* genes located close to the most significant marker ID10-194305124, which had the highest LOD score in the *qRRs-10.1* locus. Five putative *R* genes (CA10g12570, CA10g12800, CA10g13010, CA10g13020, and CA10g13030), delimited to the region 193.4–196.3 Mb (~2.9 Mb) were verified to have either an *NB-ARC* or an *LRR* domain based on sequence alignment against the NR and Swiss-Prot databases (Table S4). Among these, CA10g12570 and CA10g12800 were physically located very closely to the marker ID10-194305124. Additionally, three tandemly arrayed genes, CA10g13010, CA10g13020, and CA10g13030, were located in the physical interval of 196,298,696–196,301,165 bp, which is only ~2 Mb and ~90 Kb away from the significant markers ID10-194305124 and ID10-196208712, respectively.

Previous studies have identified *R* genes acting in tandem [53,57,58,59,60]. In pepper, potyvirus resistance locus *Pvr4* and tomato spotted wilt virus resistance locus *Tsw* are located in the same locus at the end of chromosome 10. Eight genes encoding CC-NBS-LRR proteins are clustered within the *Pvr4* locus in *C. annuum* CM334, whereas 14 *NBS-LRR* genes have been predicted within the *Tsw* locus in *C. chinense* PI159236. Sequence alignment of the *NBS* genes in the *Pvr4/Tsw* cluster revealed that eight *CC-NBS-LRR* genes in CM334 showed co-orthology with 14 *NBS-LRR* genes of PI159236 [60]. In the present study, two (CA10g13010 and CA10g13020) of the three tandem *R* genes were predicted as the disease resistance gene *Bs2*. *Bs2* belongs to the *NBS-LRR* family [61] and produces a hypersensitive response when it recognizes its cognate AvrBs2 protein, an effector protein that is highly conserved among *Xanthomonas* spp. [62]. Recently, Kunwar et al. [63] evaluated transgenic tomato expressing elongation factor tu receptor (*EFR*) from *A. thaliana* and/or *Bs2* from pepper for the management of BW and bacterial spot. Expression of *EFR* or *Bs2/EFR* in susceptible tomato significantly reduced the disease severity of BW and bacterial spot, demonstrating the potential of using *Bs2*/*EFR* for field management of these diseases. *EFR* is an important component of pathogen-associated molecular pattern (PAMP)-triggered immunity and plays an important role in plant defense by recognizing conserved PAMPs in microbes [64]. We identified one defense-related gene, CA10g12520, encoding *PR-1*, involved in “plant–pathogen interaction” (ko: 04626), which includes PAMP-triggered immunity. Furthermore, two *LURP* genes, CA10g12960 and CA10g12960, involved in “defense response” (GO: 0006952) were identified. Knoth and Eulgem [39] found that *LURP1* is required for full basal defense to *Hyaloperonospora parasitica* and resistance to this pathogen mediated by the *R* proteins *RPP4* and *RPP5*. 

In a sequence comparison of coding regions between the parents, non-synonymous variations were identified in five candidate genes, but not CA10g12520, CA10g12570, and CA10g12800 (Table S5). We cannot rule out that differential expression of these three genes between resistant and susceptible lines may underlie difference in gene activity for BW resistance. Future research is required to genetically and functionally validate the resistance mechanisms of these seven strong candidate genes for the control of *R. solanacearum* in pepper. 

## 4. Materials and Methods 

### 4.1. Grafting Experiments

Two inbred pepper lines (*C. annuum*), BVRC 25 and BVRC 1, sourced from the Beijing Vegetable Research Center (Beijing, China), were used in the grafting experiments. BVRC 1 is highly resistant to BW, whereas BVRC 25 is highly susceptible [1]. Pepper seeds were sown in 72-cell plug trays (54 cm × 28 cm × 5.5 cm; Taizhou Longji Yuanyi Cailiao, Zhejiang, China) containing a mixture of peat and vermiculite (2:1) sterilized in an autoclave (ALP, Tokyo, Japan) for 30 min. Plants were grown in a greenhouse at 28/25 °C (day/night) under a 14 h photoperiod and were fertilized weekly with Poly-Feed (1 g/L) (Haifa Chemicals, Haifa Bay, Israel). All four possible scion/rootstock combinations, i.e., BVRC 1/BVRC 1, BVRC 25/BVRC 25, BVRC 1/BVRC 25, and BVRC 25/BVRC 1, were included in the grafting experiments. Seedlings in the four-expanded-leaf stage were grafted approximately 2 cm up the cotyledon using a steep angle cut, and the graft union was secured with grafting clips. The grafted seedlings were incubated in a mist chamber with a high humidity of 80–90% at 28/25 °C (day/night) under a 14 h photoperiod for two weeks to heal. Then, successfully grafted seedlings of each combination were transferred to a greenhouse that is used only for BW disease evaluation. Three independent replicates, each with 8–10 grafted seedlings of each grafting combination, were prepared for use in subsequent experiments. 

### 4.2. Inoculum Preparation and Inoculation

To evaluate bacterial infection dynamics in planta in real time, we previously generated a bioluminescent *R. solanacearum* strain, BL-Rs7, using the vector pXX3 carrying the *luxCDABE* gene [1]. The luminescence intensity was strongly correlated with the bacterial population in planta (*R^2^* = 0.88). Inoculums preparation and root-dipping were carried out as previously described [1], with minor modifications. BL-Rs7 was streaked on Kelman’s triphenyl tetrazolium chloride agar medium and the plate was incubated at 28 °C for 60 h [65]. Mucoid, egg-shaped *R. solanacearum* colonies with pink-reddish centers were selected and cultured on NBY nutrient agar plates at 28 °C for 60 h. Bacterial suspensions were prepared in sterile distilled water and adjusted to OD_600_ = 0.10 (~1 × 10^8^ CFU/mL). The plants were carefully removed from the 72-cell flats, and the roots were rinsed in running water. The plants were inoculated by directly dipping the roots in the BL-Rs7 suspension for 10 min. For real-time imaging, the inoculated plants were transplanted into pots (8 cm × 8 cm, diameter × height) filled with sterilized perlite, and watered with 0.1× Hoagland’s nutrient solution (Hoagland and Arnon 1950) [66]. Pots were arranged in a growth chamber (28/25 °C (day/night), 14 h photoperiod).

### 4.3. Bioluminescence Imaging

Plant colonization by BL-Rs7 was determined by bioluminescence imaging using a Berthold NightSHADE CCD camera, model LB985 (Berthold Technologies GmbH & Co. KG, Bad Wildbad, Germany). Bioluminescence images were acquired as described previously [1,67]. The imaging parameters of the sensitivity/resolution setup were 4 × 4 binning and a 5 min delay in the dark imaging chamber prior to an incremental exposure time of 180 s. After imaging, the plants were replanted into the pots and placed in the growth chamber. Root-inoculated seedlings were imaged at 5, 7, 9, 10, 11, 15, and 30 dpi to monitor the movement of bacteria. The bioluminescence signal in planta was quantified using indiGo software, and the light intensity was displayed in counts per second. 

### 4.4. Genetic Population Construction

A cross was made between BVRC 25 (female parent, P_s_) and BVRC 1 (pollen donor, P_r_) to create an F_1_ generation. An F_1_ individual was self-pollinated and backcrossed to the parental lines to obtain the progenies F_2_, BC_1_P_s_, and BC_1_P_r_. All crosses were made by controlled hand pollination in the greenhouse at the Beijing Vegetable Research Center. The six progenies, including P_s_, P_r_, F_1_, F_2_, BC_1_P_s_, and BC_1_P_r_, were used to test BW resistance inheritance. The experimental design for disease evaluation assays of the six progenies is presented in Table 1. 

### 4.5. Inoculation and Disease Evaluation 

The *R. solanacearum* phylotype I strain Rs-SY1 isolated from pepper in China [1] was used in this study. Plant culture and inoculation were carried out as described above, with minor modifications. Seedlings at the six-expanded-leaf stage were root-dip inoculated with strain Rs-SY1 at 1 × 10^8^ CFU/mL for 10 min. The inoculated plants were planted into individual 10 cm × 10 cm (diameter × height) pots containing sterilized peat and vermiculite. The pots were arranged in the greenhouse used for BW disease evaluation only. The temperature ranged from 25 to 30 °C (08:00 to 20:00) and 20 to 25 °C (20:00 to 08:00). Water and fertilizer were provided as needed. Once the susceptible parent began to exhibit disease symptoms (typically, 7 dpi), all plants were scored for disease severity.

**Figure 5:**
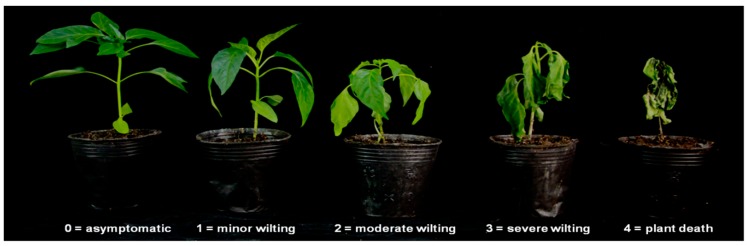
Disease severity of bacterial wilt in pepper.

At 7, 10, 15, and 20 dpi, the disease severity of each plant was scored as follows: 0 = asymptomatic; 1 = minor symptoms with less than 20% wilted leaves; 2 = moderate symptoms with 20–50% wilted leaves; 3 = severe symptoms with 50–80% wilted leaves; 4 = dead plant, 80–100% wilted leaves (Figure 5). The DSI was calculated as follows:

DSI = ∑(disease score × NDP)/N(1)

where NDP is the total number of plants with the specified disease score, and N is the total number of plants. The area under the disease progress curve (AUDPC) was calculated using the agricolae R package [68] based on the assessed plant disease score. The disease response was based on the disease score at 20 dpi; a plant with a DSI of ≤2 was considered healthy, and wilting was defined as a DSI of >2. 

### 4.6. DNA Extraction 

Young leaves were collected from P_s_, P_r_, F_1_, and 440 F_2_ plants at the four-expanded-leaf stage. Genomic DNA was extracted by the cetyltrimethylammonium bromide–chloroform method, as described by Fulton et al. [69], with minor modifications. DNA integrity was evaluated by 1.5% (*w*/*v*) agarose gel electrophoresis, and the concentration was determined using a NanoDrop 2000 spectrophotometer (Thermo Fisher Scientific, Wilmington, DE, USA). Final DNA concentrations were adjusted to 150 ng/μL with a total DNA amount of at least 25 μg/plant. 

### 4.7. SLAF Library Construction and Sequencing 

Two DNA pools were constructed for SLAF-seq analysis, namely, an R-pool (resistant to BW) and an S-pool (susceptible to BW). The pools were generated by mixing equal amounts of DNA extracted from 29 resistant (DSI = 0 at 20 dpi) F_2_ individuals and 29 of the most susceptible (DSI = 4 at 7 dpi) F_2_ individuals. Genomic DNA from both parents and the two DNA pools was digested with the restriction enzyme *Hae*III (New England BioLabs, Ipswich, MA, USA). A single-nucleotide A overhang was added to the digested fragments using the Klenow DNA polymerase fragment (New England Biolabs, Ipswich, MA, USA) and dATP at 37 °C. Then, polyacrylamide gel electrophoresis-purified duplex tag-labeled sequencing adapters [70] were ligated to the A-tailed DNA with T4 DNA ligase (New England BioLabs, Ipswich, MA, USA). The reaction products were pooled and purified using QIAquick spin columns (Qiagen, Hilden, Germany). The purified products were electrophoresed on a 2% agarose gel, and fragments with sizes of 414–514 bp were collected as SLAFs and purified using a gel extraction kit (Qiagen, Hilden, Germany). The purified SLAFs were sequenced on a HiSeq 2500 platform (Illumina, San Diego, CA, USA) at Biomarker Technologies Corporation in Beijing, China (http://www.biomarker.com.cn).

### 4.8. SLAF-seq Data Analysis

SLAFs grouping and genotyping were carried out as described previously [28]. Briefly, after filtering out the low-quality reads (quality score <30e), the clean SLAF pair-end reads were clustered based on sequence similarity using BLAST (tilesize = 10, stepsize = 5). The pair-end clean reads were mapped onto the *C. annuum* cv. CM334 reference genome (v.1.55; ftp://ftp.solgenomics.net/genomes/Capsicum_annuum/C.annuum_cvCM334/assemblies/) using the Burrows–Wheeler alignment tool with default parameters [71]. SNPs were detected using the GATK software [35] per the user guide (https://www.broadinstitute.org/gatk/guide/best-practices.php). To ensure the accuracy of the SNPs identified using GATK, SAMtools software was also used to detect SNPs [36]. SNPs that were detected by both GATK and SAMtools were selected for further analysis. Prior to association analysis, the SNPs were filtered using the following criteria: SNPs with multiple alleles, SNPs with a sequencing depth of less than 4× in each pool or parent, SNPs with the same genotypes among pools, and SNPs with recessive alleles that were not inherited from parents with recessive genotypes in the pools were all filtered out. Ultimately, a collection of high-quality SNP markers was obtained for use in association analysis.

### 4.9. Association Mapping Analysis

Association mapping was performed using both the Euclidean distance (ED) [37] and the SNP index algorithm [26]. The ED between the allele frequencies at each SNP in the R- and S-pools was calculated as in Hill et al. [37], using the equation

(2) $$ED=\sqrt{{\left(A_{\mathrm{mut}}-A_{\mathrm{wt}}\right)}^{2}+{\left(C_{\mathrm{mut}}-C_{\mathrm{wt}}\right)}^{2}+{\left(G_{\mathrm{mut}}-G_{\mathrm{wt}}\right)}^{2}+{\left(T_{\mathrm{mut}}-T_{\mathrm{wt}}\right)}^{2}},$$

where each letter (A, C, G, T) indicates the frequency of the corresponding DNA nucleotide. The ED values were squared to decrease the effects of noise and increase the effects of large EDs. Data were then fitted using LOESS regression, and the threshold for the significance of marker–trait associations was set at median + 3SD of the LOESS-fitted values [37]. The genome regions at which the LOESS-fitted values exceeded the threshold were designated as candidate regions associated with resistance to BW in pepper.

Association analysis method based on the SNP index allows finding significant differences in genotype frequency between the pools, indicated by Δ(SNP index) [26]. In this SLAF-seq project, M stands for BVRC 25 (BW-susceptible parent) and P stands for BVRC 1 (BW-resistant parent), and aa denotes the genotype of the BW-susceptible parent, and ac denotes the genotype of the BW-resistant parent. The SNP index indicates the proportion of reads with SNPs that are different from the reference sequence. The Δ(SNP index) was calculated as follows:

SNP index (aa) = Maa/(Paa + Maa),(3)

SNP index (ac) = Mac/(Pac + Mac),(4)

Δ(SNP index) = SNP index (aa) – SNP index (ac),(5)

where Maa indicates the depth of the aa population derived from M; Paa indicates the depth of the aa population derived from P; Mac indicates the depth of the ac population derived from M; Pac indicates the depth of the ac population derived from P. Δ(SNP index) = 1 if the bulked DNA comprise only the parent BVRC 1; Δ(SNP index) = −1 if it comprised only the parent BVRC 25; Δ(SNP index) = 0 if the bulked DNA has the same SNP index as both parents in the genome region. The average SNP index was plotted against the position of each sliding window (4 Mb) in the CM334 genome. The Δ(SNP index) data were then fitted using LOESS regression, and the threshold for the significance of marker–trait associations was set at 1% of the largest Loess-fitted values [26]. The regions for which Δ(SNP index) values exceeded the threshold were considered candidate regions (potential QTLs) associated with resistance to BW. Finally, ED, SNP index, and ΔSNP index values were plotted, and the intersections between candidate regions identified using the ED and SNP index methods were designated as final candidate BW resistance associated regions. 

### 4.10. Insertion/Deletion (InDel) and SNP Marker Development 

The regions associated with resistance to BW identified by SLAF-BSA were verified by polymorphic SNP and InDel marker-based traditional QTL analysis. To widely characterize the sequence polymorphisms between the parents and within the candidate regions, we subjected the two parental lines to whole-genome resequencing with an average depth of ~31.65 on the Illumina X Ten platform at Shanghai Majorbio Bio-Pharm Technology (Shanghai, China). 

The raw reads were filtered using Trimmomatic [71]. The clean reads were mapped to the reference genome CM334 version 1.55 using Burrows–Wheeler alignment software [72], and InDels and SNPs were identified using the GATK program [35] per the user guide (https://www.broadinstitute.org/gatk/guide/best-practices.php). 

Primers for the markers were designed with Primer 5.0 software (http://www.PromerBiosoft.com), and were named based on the physical positions of pepper reference genome CM334 (v.1.55). The markers were initially evaluated in the parental lines to find clear polymorphisms, and the polymorphic markers were genotyped in the 440 individuals of the F_2_ mapping population. The polymorphic InDel and SNP primers used for QTL mapping are listed in supplementary Table S1. 

### 4.11. Genotyping of the Population

PCR analysis of InDels was carried out in 10 μL reactions containing 1 μL of DNA (15 ng/μL), 0.5 μL each of the forward and reverse primers (10 μM), 3 μL of ddH_2_O, and 5 μL of GoTaq Green Master Mix (Promega, Madison, WI, USA). The thermal cycling conditions were as follows: initial denaturation at 94 °C for 3 min, 38 cycles of denaturation at 94 °C for 30 s, annealing at 52 °C for 30 s, and extension at 72 °C for 30 s, and a final extension at 72 °C for 5 min. For each InDel marker with a small variant sequence (6–10 bp), the PCR products were separated on 6% nondenaturing polyacrylamide gels at 150 V for 1 h. After silver staining, the gel was observed with a film illuminator for band analysis. For each InDel marker with a large variant sequence (21–65 bp), the PCR products were separated on 4% agarose gels, stained with ethidium bromide, and photographed using a GIAS-4400 gel documentation system (Beony Science and Technology, Beijing, China). For each SNP, two allele-specific forward primers and one common reverse primer were used for genotyping with kompetitive allele-specific PCR (KASPar) assays as described by Su et al. [73]. KASPar assays were carried out using 1 μLreactions in 1536-well microplates (KBS-0751-001, KBioscience, Hoddesdon, UK), 12 nM of each of the allele-specific forward primers, 30 nM of the reverse primer, and 4 ng of genomic DNA. PCRs were run in a GenePro thermal cycler (Hydrocycler, Hoddesdon, UK) using the following cycling conditions: 15 min at 94 °C, 10 touchdown cycles of 20 s at 94 °C and 60 s at 65–57 °C (reduction of 0.8 °C/cycle), and 26–42 cycles of 20 s at 94 °C and 60 s at 57 °C. Fluorescence was detected using an omega FLUOstar scanner (BMG Labtech, Ortenberg, Germany), and the data were analyzed using KlusterCaller 1.1 software (KBioscience, Hoddesdon, UK). Detailed instructions can be downloaded at www.kbioscience.co.uk. 

### 4.12. Linkage Map Construction and QTL Mapping 

The genetic map was constructed using MAPMAKER/EXP v. 3.0 [74]. The genotyping data and disease severity values (DSI and AUDPC) for the 440 F_2_ plants were used for the QTL analysis. CIM [75] was used to identify QTL-related markers, using WinQTLCart 2.5 software [76]. To obtain more accurate results, LOD score of 3.0 was used as the threshold for testing the significance of the QTL peaks with 1 000 permutations and a significance level of *p* < 0.05. The phenotypic variance explained by each marker was calculated as an *R^2^* value using single marker analysis. The software package R/qtl [77] was also used to verify the QTLs. The dominance ratio (DR) was estimated from the CIM analysis results. DR = 2|d/a| [78,79], where a and d are the additive and dominance estimates, respectively. DR is used to describe the gene effect: DR < 0.2, additive; 0.2 < DR < 0.8, partially dominant; 0.8 < DR < 1.2, dominant; DR > 1.2, overdominant. 

### 4.13. Annotation of the Predicted Genes and Sequence Variants in the QTL Region

The predicted genes closer than ±2 Mb from the highest significant marker were identified based on the annotation of the pepper CM334 reference genome (v. 1.55) [80]. Putative functions of the predicted genes were further annotated based on sequence alignment with the NCBI nonredundant protein database (NR, ftp://ftp.ncbi.nih.gov/blast/db/), SwissProt (http://www.uniprot.org/), Pfam (http://pfam.xfam.org/), Gene Ontology (GO, http://www.geneontology.org/) [81], Kyoto Encyclopedia of Genes and Genomes (KEGG, http://www.genome.jp/kegg/) [82], and Clusters of Orthologous Groups of proteins (COG, http://www.ncbi.nlm.nih.gov/COG/) [83] databases by BLAST, with default parameters. Sequence variants in the target QTL were identified by comparison of the resequencing data of the parents against the pepper genome of CM334 (v.1.55), as described above. The localizations and coding effects of SNPs closed to the predicted genes were annotated using SnpEff software (http://snpeff.sourceforge.net/SnpEff_manual.html) [84].

## 5. Conclusions

Analysis of the dynamics of bioluminescent *R. solanacearum* colonization in reciprocal grafts revealed that the resistant BVRC 1 rootstock effectively suppressed the spreading of bacteria into the scion, indicating that BVRC 1 is a good rootstock for the management of BW in pepper. SLAF-BSA combined with QTL mapping identified a major BW resistance QTL, *qRRs-10.1*, on chromosome 10 of the pepper line BVRC 1. We found five predicted *R* genes and three defense-related genes to be strong candidate genes for *qRRs-10.1* that are potentially involved in BW resistance. All of them are located in close proximity to the significant marker ID10-194305124 or ID10-196208712. Therefore, these two adjacent InDel markers could be useful for tracing down resistance alleles of the eight candidate genes, and for MAS of BW resistance in pepper.

## Figures and Tables

**Table 1:** Disease evaluation design and the values of the wilt rate, DSI, and AUDPC for parents BVRC 25 and BVRC 1 and their progenies in response to root-dip inoculation with *R. solanacearum* strain Rs-SY1.

Generations ^a^	Experimental Design for Disease Evaluation Assays			Resistance Components		
	Test × Block × Plants	No. of Plants	Inoculation Time	DSI ^b^	Wilt Rate (%) ^c^	AUDPC ^d^
P_s_	3 × 1 × 20 − 25	68	Summer of 2016	3.98 ± 0.04	100	42.92 ± 1.84
			Autumn of 2016			
			Summer of 2017			
P_r_	3 × 1 × 15 − 25	66	Summer of 2016	0.31 ± 0.24	4.58 ± 3.95	1.68 ± 1.28
			Autumn of 2016			
			Summer of 2017			
F_1_ (♀P_s_ × ♂P_r_)	3 × 1 × 15 − 25	68	Summer of 2016	1.76 ± 0.17	48.59 ± 5.38	14.56 ± 3.08
			Autumn of 2016			
			Summer of 2017			
BC_1_P_s_	1 × 1 × 31	31	Summer of 2017	2.71	71.97	23.08
BC_1_P_r_	1 × 1 × 33	33	Summer of 2017	0.86	24.94	7.2
F_2_	1 × 1 × 440	440	Autumn of 2016	1.88	47.78	18.36

^a^ P_s_ and P_r_ are the susceptible parent line BVRC 25 and resistant parent line BVRC 1, respectively. ♀ and ♂ represent female and male parent, respectively. ^b^ The disease severity index (DSI) was calculated at 20 days after root-dip inoculation based on a 0 to 4 rating scale as follows: DSI = ∑(disease score × number of plants with each disease score)/total number of plants. ^c^ Wilting was defined as DSI at 20 dpi of >2. ^d^ The area under the disease progress curve (AUDPC) was calculated from scores (0–4) evaluated at 7, 10, 15, and 20 dpi.

**Table 2:** Common bacterial wilt (BW) resistance-associated regions were estimated based on the SNP index and by Euclidean distance analysis.

Chr ID	Start Position (bp)	End Position (bp)	Interval Size (Mb)
Pepper.v.1.55 Chr10	56,910,000	69,110,000	~12.20
Pepper.v.1.55 Chr10	111,090,000	183,670,000	~72.58

**Table 3:** Genetic mapping information of markers within the *qRRs-10.1* QTL region mapped at 20 dpi with *R. solanacearum* strain Rs-SY1.

InDel Markers ^a^	Genetic Distance (cM)	Composite Interval Mapping Results					Mean AUDPC of Three Genotypic Classes in F_2_ Plants ^g^		
		LOD ^b^	*R^2^* (%) ^c^	A ^d^	D ^e^	DR ^f^	B:B	A:A	A:B
ID10-178408813	63.6	7.47	13.65	0.81	−0.21	0.52	7.30	33.80	16.87
ID10-182178715	64.9	8.12	14.00	0.86	−0.20	0.46	7.69	33.97	17.71
ID10-185236974	66.8	8.37	17.82	0.94	−0.17	0.37	7.12	35.00	17.60
ID10-186260160	66.5	8.37	17.82	0.87	−0.21	0.47	7.04	34.91	17.74
ID10-190060186	66.6	8.38	17.82	0.87	−0.21	0.47	7.12	34.91	17.74
ID10-194305124	71.0	9.93	19.01	0.97	−0.10	0.21	6.36	35.52	18.05
ID10-196208712	72.3	3.59	18.41	0.73	−0.13	0.35	7.45	35.55	17.85

^a^ Markers were named based on the physical positions in reference genome CM334 (v.1.55). ^b^ LOD, log-likelihood. ^c^ *R^2^* (%), percentage of variation explained by each maker. ^d^ A, value of the additive effect of each maker. ^e^ D, value of the dominance effect of each marker. A negative sign indicates that the increasing resistance of the QTL alleles was contributed by the resistant parent. ^f^ 2|d/a|, dominance ratio (DR), with a and d being the additive and dominance estimates, respectively. DR < 0.2 (additive), 0.2 < DR < 0.8 (partially dominant), 0.8 < DR < 1.2 (dominant), DR >1.2 (overdominant). ^g^ AUDPC, area under the disease progress curve. Lower mean AUDPC was associated with greater plant disease. B:B, allele of resistant line BVRC 1; A:A, allele of susceptible line BVRC25; A:B, heterozygous genotype.

**Table 4:** Resistance and defense-related genes identified within the major QTL, *qRRs-10.1*.

Gene ID	Functional Annotation	Database ^a^	No. of Non-Synonymous in Coding Region
CA10g12520	Pathogenesis-related protein (PR-1)	NR; Swiss-Prot; KEGG: plant–pathogen interaction;	0
CA10g12570	Tospovirus resistance protein E	CM334 (v.1.55); COG; Swiss-Prot	0
CA10g12800	Putative disease resistance protein	CM334 (v.1.55); Pfam; Swiss-Prot	0
CA10g12960	protein LURP-one-related 15-like	CM334 (v.1.55); Nr, Swiss-Prot; Pfam; GO: defense response;	2
CA10g12980	protein LURP-one-related 15-like	CM334 (v.1.55); Nr, Swiss-Prot; Pfam, GO: defense response;	1
CA10g13010	Disease resistance protein *BS2*	CM334 (v.1.55); NR	8
CA10g13020	Disease resistance protein *BS2*	CM334 (v.1.55); Pfam; Swiss-Prot	7
CA10g13030	NBS-coding resistance gene analog	CM334 (v.1.55); NR	5

^a^ NR, NCBI nonredundant protein database; GO, Gene Ontology; KEGG, Kyoto Encyclopedia of Genes and Genomes.

## Supplementary Materials

Supplementary Materials can be found at https://www.mdpi.com/1422-0067/20/23/5887/s1.

## Abbreviations

QTL	quantitative trait locus
SNP	single-nucleotide polymorphism
InDel	insertion/deletion
SLAF	specific-locus amplified fragment
BSA	bulked segregant analysis
ED	Euclidean distance
BW	bacterial wilt
CIM	composite interval mapping
DSI	disease severity index
dpi	days post-inoculation
AUDPC	area under the disease progress curve
LOD	log-likelihood
NGS	next-generation sequencing
DR	dominance ratio
GO	gene ontology
KEGG	kyoto encyclopedia of genes and genomes
COG	cluster of orthologous groups of proteins
KASPar	kompetitive allele-specific PCR
LRR	leucine-rich repeat
NB	nucleotide binding
PAMP	pathogen-associated molecular pattern
MAS	marker-assisted selection
